# Several domains from VAR2CSA can induce *Plasmodium falciparum *adhesion-blocking antibodies

**DOI:** 10.1186/1475-2875-9-11

**Published:** 2010-01-11

**Authors:** Ali Salanti, Mafalda Resende, Sisse B Ditlev, Vera V Pinto, Madeleine Dahlbäck, Gorm Andersen, Tom Manczak, Thor G Theander, Morten A Nielsen

**Affiliations:** 1 Centre for Medical Parasitology at Department of International Health, Immunology and Microbiology, University of Copenhagen and Department of Infectious Diseases, Copenhagen University Hospital (Rigshospitalet), Copenhagen, Denmark

## Abstract

**Background:**

Malaria caused by *Plasmodium falciparum *can result in several different syndromes with severe clinical consequences for the about 200 million individuals infected each year. During pregnancy, women living in endemic areas become susceptible to malaria due to lack of antibodies against a unique *P. falciparum *membrane protein, named VAR2CSA. This antigen is not expressed in childhood infections, since it binds chondroitin sulphate A (CSA) expressed on the intervillous space in the placenta. A vaccine appears possible because women acquire protective antibodies hindering sequestration in the placenta as a function of parity. A challenge for vaccine development is to design small constructs of this large antigen, which can induce broadly protective antibodies. It has previously been shown that one domain of VAR2CSA, DBL4-FCR3, induces parasite adhesion-blocking antibodies. In this study, it is demonstrated that other domains of VAR2CSA also can induce antibodies with inhibitory activity.

**Methods:**

All VAR2CSA domains from the 3D7 and HB3 parasites were produced in *Baculovirus*-transfected insect cells. Groups of three rats per protein were immunized and anti-sera were tested for surface reactivity against infected erythrocytes expressing FCR3 VAR2CSA and for the ability to inhibit FCR3CSA parasite adhesion to CSA. The fine specificity of the immune sera was analysed by VAR2CSA peptide arrays.

**Results:**

Inhibitory antibodies were induced by immunization with DBL3-HB3 T1 and DBL1-3D7. However, unlike the previously characterised DBL4-FCR3 response the inhibitory response against DBL1-3D7 and DBL3-HB3 T1 was poorly reproduced in the second rounds of immunizations.

**Conclusion:**

It is possible to induce parasite adhesion-blocking antibodies when immunizing with a number of different VAR2CSA domains. This indicates that the CSA binding site in VAR2CSA is comprised of epitopes from different domains.

## Background

Pregnancy associated malaria (PAM) causing maternal anaemia, low birth weight and stillbirth, is a severe manifestation of *Plasmodium falciparum *infection [1]. PAM is caused by infected erythrocytes (IE) that sequester in the intervillous space of the placenta [2]. The ability to sequester in the vascular bed whereby the parasite avoids immune mechanisms in the spleen is a hallmark of the particular virulence of *P. falciparum*. The IE bind host receptors on various endothelia through antigens called *P. falciparum *erythrocyte membrane protein 1 (PfEMP1). The PfEMP1 protein family, encoded by the *var *genes, is constituted of large proteins of 150-350 kDa with several Duffy-binding-like (DBL) domains. Different PfEMP1 molecules have different receptor specificities, and clonal switching between expression of the various *var *gene products, in a mutually exclusive manner, allows the parasite to modify its adhesion properties accordingly (reviewed in [3]). There are about 60 copies of highly different *var *genes in each parasite genome and a high sequence variation among genomes [4-6]. Expression of different PfEMP1 variants allow the parasites to escape previously acquired antibody responses, and clinical protection occurs when a large repertoire of variant specific antibodies allows the host to control the infection [7]. After repeated infections during childhood in endemic areas the acquired repertoire of antibodies against the variant surface antigens will progressively protect against variants expressed during severe, mild and asymptomatic infections [8,9]. During pregnancy the parasite again escapes acquired immunity by expressing variants not encountered during childhood disease. This is mediated by parasites occupying a new niche, the developing placenta. The interaction between parasite antigens on the surface of the IE and chondroitin sulphate A (CSA) in the placenta is one of the most direct associations between binding phenotype and disease outcome in *falciparum *malaria. Placental parasites and parasite lines selected for CSA binding *in vitro *express a unique *var *gene named *var2csa *[10,11]. VAR2CSA is expressed on the surface of IE panned on CSA and on IE isolated from infected placentas [12,13] and parasite clones where the *var2csa *gene is disrupted lose the ability to bind CSA [14]. Several domains and regions of VAR2CSA have been shown to bind CSA *in vitro*, however, the specificity of single VAR2CSA domain binding to CSA does not seem to be exclusive for CSA type glycans [15-18].

Women in malaria endemic areas acquire antibodies that protect against PAM as a function of parity [1]. The mechanism of protection is suggested to be based on antibodies that block binding of IE to CSA [19]. Likewise, high anti-VAR2CSA IgG levels are correlated with protection against the clinical consequences of PAM [13]. These findings suggest that it is feasible to develop a VAR2CSA-based vaccine to protect women in malaria endemic areas against PAM. A challenge for vaccine development is to define VAR2CSA constructs of a size compatible with protein-vaccine production, which elicit pan-reactive antibodies that abrogate binding of parasites in the placenta. It has previously been shown that antibodies induced against DBL6-FCR3 partially inhibited parasite binding and that this inhibitory activity was only present in serum collected during the immunization but absent in the final bleed [20]. Recently, it was shown that DBL4-FCR3 induced a broadly IgG based parasite adhesion-blocking response, which increased during the immunizations and was highly reproducible in subsequent immunizations with the same antigen [21]. These DBL4 antibodies are currently being tested for the capacity to inhibit binding to CSA in a large panel of placental parasites. It is possible that the DBL4-FCR3 antibodies do not inhibit all isolates due to sequence variation within VAR2CSA. In that case an effective PAM vaccine would need to be based on multiple VAR2CSA serotypes. The aim of this study was to define additional DBL domain, which can induce parasite adhesion-blocking antibodies in order to make a multidomain vaccine, which presumable could protect women against multiple *P. falciparum genotypes*.

Two domains, DBL1-3D7 and DBL3-HB3 T1 were shown to induce a cross-reactive inhibitory response, however, in line with the published data on DBL6-FCR3 the inhibitory response was either not sustained during the entire immunization protocol or was not reproducible.

## Methods

### Plasmodium falciparum cultures

Parasite culture was grown as previously described [22]. In brief, parasites were maintained in culture using 5% haematocrit of human blood group 0+ blood in parasite medium consisting of RPMI-1640 supplemented with 25 mmol/l sodium bicarbonate (Sigma-Aldrich), 0.125 μg/ml gentamycin, 0.125 μg/ml Albumax II (Invitrogen) and 2% normal human serum. To select for VAR2CSA expression, IE were repeatedly panned on BeWo-cells. All isolates were mycoplasma negative and were regularly genotyped using nested GLURP and MSP-2 primers in a single PCR step.

### Protein production

All VAR2CSA constructs were based on native *var2csa *and cloned from genomic parasite DNA. Control constructs were cloned from DNA from either FCR3 var1-DBL3γ or 3D7 PF08_0141-DBL2β. Gene fragments were cloned into the *Baculovirus *vector, pAcGP67-A (BD Biosciences) modified to contain the V5 epitope upstream of a histidine tag in the C-terminal end of the constructs. Linearized Bakpak6 *Baculovirus *DNA (BD Biosciences) was co-transfected with pAcGP67-A into Sf9 insect cells for generation of recombinant virus particles. Histidine-tagged recombinant protein was purified on Ni^2+ ^sepharose columns from the supernatant of *Baculovirus *infected High-Five insect cells using an ΔKTAxpress purification system (GE-Healthcare).

### Rat immunizations

Wistar rat anti-sera raised against the VAR2CSA domains from HB3 and 3D7 were produced by injection of 40 μg of recombinant protein in Freund's complete adjuvant, followed by two booster injections of 40 μg of protein in Freund's incomplete adjuvant at 2 1/2-week intervals. Anti-sera were collected eight days after the final boosting injection. All immunizations induced antibodies against the recombinant proteins as measured by Enzyme-Linked Immunosorbent Assay (ELISA) of the final bleed. Immunizations with the DBL3-HB3 T1 protein were done five times and six bleeds were collected. The endpoint titer of selected serum samples and IgG were determined by ELISA. All procedures regarding animal immunizations complied with European and national regulations.

### Flow cytometry and binding inhibition assay

Flow-cytometry was used to test the reactivity of rat serum to VAR2CSA on the surface of the IE. In brief, parasite cultures were enriched to contain late trophozoite and schizont stage parasites by exposure to a strong magnetic field. Aliquots (2 × 10^5 ^IE) were labelled by ethidium bromide and sequentially exposed to 10 μl rat serum and 1 μl anti-rat IgG-FITC (Zymax, Invitrogen). Data was acquired using a FC500 flow-cytometer (Beckman Coulter). All samples relating to a particular parasite isolate were processed and analysed in a single assay. Parasite binding assays were done as described in [21]. Briefly, 2 × 10^5 ^tritium labelled late-stage IE and 15 μl rat serum or IgG in a total volume of 100 μl were added in quadruplicates to wells coated with 2 μg/ml of the commercially available chondroitin sulphate proteoglycan (CSPG) decorin (D8428, Sigma-Aldrich). The binding to decorin was abrogated by soluble CSA (C9819, Sigma-Aldrich) and chondroitinase treatment. After incubation for 30 min at 37°C unbound IE were washed away by resuspension performed by a pipetting robot (Beckman Coulter). The proportion of adhering IE was determined by liquid scintillation counting on a Topcount NXT (Perkin-Elmer).

### Pepscan analysis

A peptide array containing 442 31mer peptides corresponding to the extracellular part of 3D7 VAR2CSA was used for antibody binding studies [23]. The sequences of the peptides had an overlap of six residues and the purity of the peptides was expected to be 70% or higher. The peptides were synthesized with a cysteine at amino acid position 15 allowing some secondary structure. Structural modelling and mapping of residues was done as described [23].

## Results

### Cloning and expression of VAR2CSA DBL proteins

To identify other domains than the published DBL4-FCR3 [21], which could induce inhibitory antibodies, all single domains from 3D7 VAR2CSA and HB3 VAR2CSA were cloned (Table 1). Published data on rabbit antibodies induced against all 3D7 VAR2CSA DBL domains showed that these antibodies reacted with IE [24] but failed to inhibit parasite binding (data not shown). The 3D7 VAR2CSA DBL domains were this time cloned and expressed with the same border definitions as recently presented for the FCR3 domains [21]. The HB3 parasite genome harbours two different *var2csa *genes (T1 and T2) and both variants were cloned and expressed. However, the DBL2 T2 and the DBL6 T2 did not express in sufficient amounts for immunizations and thus these two domains were not included in the analysis (Table 1).

**Table 1:** List of immunogens. 3D7 and HB3 VAR2CSA constructs.

3D7 VAR2CSA single-domains	First and last amino acid in 3D7 VAR2CSA
DBL1	57-437
DBL2	542-871
DBL3	1216-1571
DBL4	1552-1942
DBL5	1984-2331
DBL6	2313-2642
**HB3 VAR2CSA Single-domains**	**First and last amino acid in HB3 VAR2CSA**
DBL1 T1	58-403
DBL1 T2	58-399
DBL2 T1	543-901
DBL2 T2	Not Expressed
DBL3 T1	1224-1615
DBL3 T2	1213-1601
DBL4 T1	1584-1964
DBL4 T2	1598-1986
DBL5 T1	2031-2356
DBL5 T2	2031-2338
DBL6 T1	Not Expressed

### Reactivity of rat IgG against native VAR2CSA and capability to block parasite adhesion to CSA

**Figure 1:**
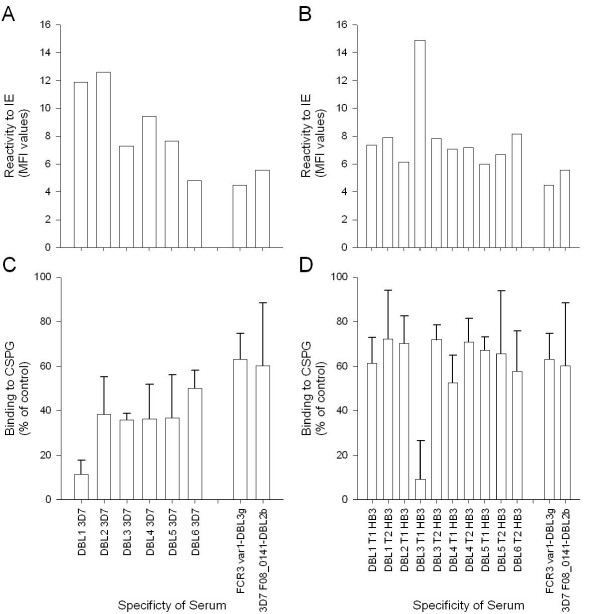
**Surface reactivity and inhibitory capacity of serum specific for DBL domains of VAR2CSA from 3D7 and HB3**. **A and B**: Sera from three rats immunized with the same recombinant protein were pooled and tested in FACS. The surface reactivity was measured using 10 μl serum in a total volume of 100 μl against 2 × 10^5 ^late trophozoite and schizont-infected erythrocytes (IE). To measure the level of rat IgG bound to the surface of the IE 1 μl rabbit anti-rat IgG-FITC was used in a total volume of 100 μl and the mean fluorescence intensity of 5000 cells was analysed and shown in white bars. The assays were performed twice with similar results. **C & D**: For inhibition of IE binding to CSPG, 15 μl of rat serum in a total volume of 120 μl were tested in triplicate wells of 96 well Falcon plates coated with 2 μg/ml CSPG. Serum pools and tritium labelled parasites (2 × 10^5^) were added simultaneously to the wells and incubated for 2 hours. Hereafter the plate was washed using a pipetting robot and the remaining cells were harvested on a filter plate and counted using scintillation. The mean percentage of binding is shown relative to binding in wells without inhibitor. Error bars are standard deviations of triplicate measurements. The assays were performed twice with similar results. In both types of assays serum from rats immunized with FCR3 var1-DBL3γ and 3D7 PF08_0141-DBL2 β were used as negative controls.

The VAR2CSA proteins were used to immunize groups of three rats. It was not possible to test inhibition of 3D7 binding, since this parasite binds poorly to CSA. With regard to the HB3 parasite, this strain harbours two variants of *var2csa *in the genome and panning on CSA results in expression of both *var2csa *variants. Therefore, all sera were evaluated on the heterologous and strongly CSA adhering parasite strain, FCR3CSA. Accordingly only cross-reactive antibodies are measured, which, in essence correspond to the response one would want from a given vaccine. All 3D7 proteins except DBL6 induced cross-reactive IgG reacting with native VAR2CSA on the surface of IE. DBL1 and DBL2 induced the highest reacting IgG (Figure 1A). The HB3 proteins induced generally poorly cross-reactive antibodies with the exception of DBL3 T1 with a MFI value of 15 (Figure 1B). The DBL5 domain has in several publications been shown to induce strong and cross-reactive IgG, however, the three different DBL5 domains produced here induced IgG, which reacted poorly with FCR3CSA (Figure 1A and 1B). The sera were tested for the ability to inhibit FCR3CSA binding to CSA. Interestingly, the DBL1-3D7 and DBL3-HB3 T1 sera had inhibitory activity and almost completely blocked the parasite adhesion to CSA (Figure 1C and 1D, respectively). None of the other HB3 proteins elicited inhibitory antibodies when comparing to the negative control sera, whereas all the other 3D7 DBL domain sera appeared to have a weak adhesion-interfering effect.

### Reproducibility of the inhibitory response

**Figure 2:**
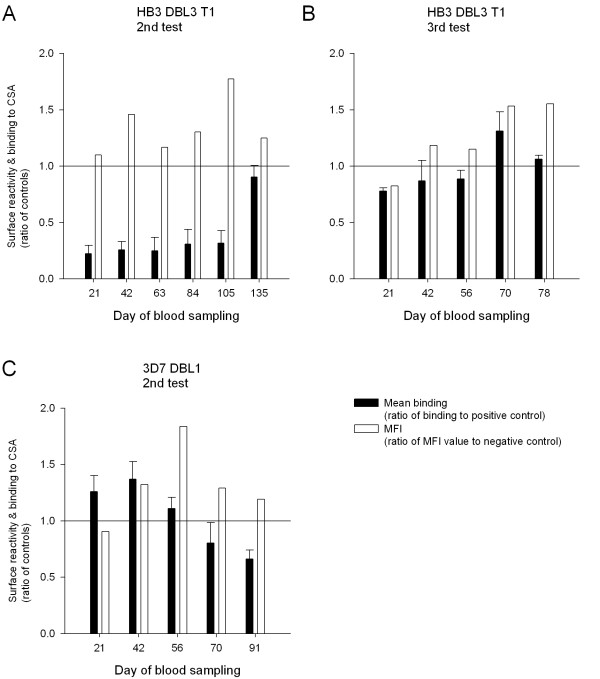
**Surface reactivity and inhibitory capacity of serum from rats immunized again with DBL3-HB3 T1 and DBL1-3D7**. Surface reactivity (white bars) and inhibition of binding (black bars) assays were performed as described in Figure 1. White bars represent the ratio between the MFI of a given sample divided by MFI of the negative control. Black bars represent the ratio between the counts per minute (CPM) in a given sample divided by the CPM of the positive control. Error bars indicate standard deviations. **A **and **B**: Pools of sera from consecutive blood sampling from rats immunized with DBL3-HB3 T1. **C **Pools of sera from consecutive blood sampling from rats immunized with DBL1-3D7. The assays were performed twice with similar results.

To further analyse the inhibitory response induced by DBL3-HB3 T1 and DBL1-3D7 two groups of rats were immunized and the different bleeds during the immunization procedure were analysed. The rats were immunized twice before the first bleed was collected at day 21. The sera were tested in two different assays measuring the capacity to react with native VAR2CSA on IE and for the capacity to inhibit parasite binding to CSA *in vitro*. The first bleed from the DBL3-HB3 T1 immunizations blocked parasite adhesion to CSA but did not stain the surface of the parasites (Figure 2A). The inhibition remained at the same level for the subsequent four bleeds, whereas the surface reactivity increased. However, at the final bleed, taken 40 days after the last immunization, the IgG remained immuno fluorescence assay (IFA) positive, but no inhibitory effect was detected. In a repeated immunization experiment three new rats were immunized with DBL3-HB3 T1. This time, adhesion-blocking antibodies were not detected in any of the bleeds, whereas the IgG reactivity against FCR3CSA increased during the immunizations (Figure 2B). When repeating the DBL1-3D7 immunizations, there was no appreciable anti-adhesive effect of the serum in any of the bleeds. Only the third bleed appeared to react with the surface of FCR3CSA, although this reactivity was not present in the final bleed (Figure 2C).

### Epitope mapping of induced anti-VAR2CSA IgG

**Figure 3:**
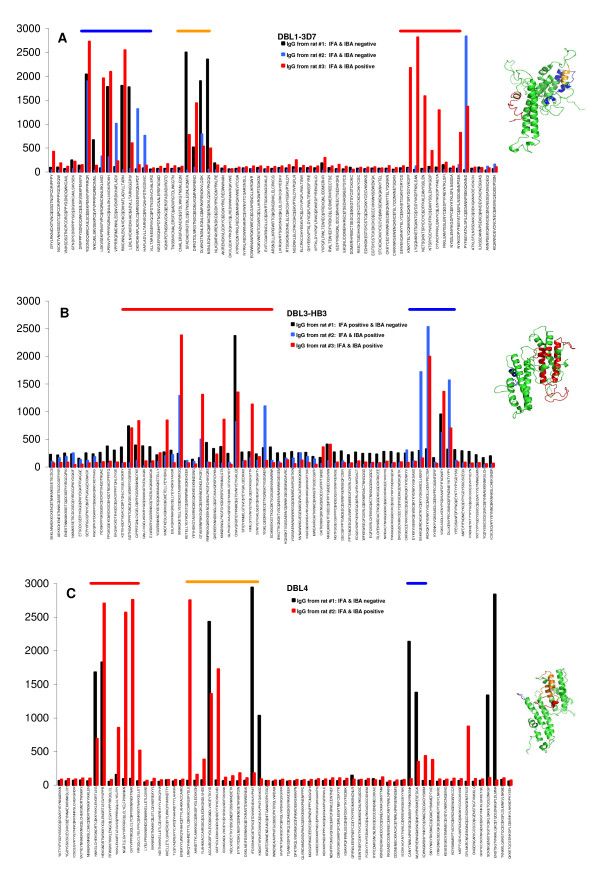
**Fine epitope mapping of anti-VAR2CSA IgG**. The fine specificity of the antibody response in inhibitory and non-inhibitory sera was analysed using a peptide array covering the extracellular part of 3D7 VAR2CSA. Inhibitory rat sera are shown with red vertical bars and non-inhibitory sera with black or blue vertical bars. IgG from three individual rats immunized with DBL1-3D7 **(A) **and DBL3-HB3 T1 **(B) **were analysed and IgG from two individual rats immunized with DBL4-FCR3 (red bars) and DBL4-3D7 (black bars) **(C)**. B-cell epitopes are marked on the graph with horizontal bars in blue, red or orange and were mapped on a structural model of the corresponding domain based on the 3D7 sequence.

The fine specificity of the antibody response in inhibitory and non-inhibitory sera was analysed using a peptide array covering the extracellular part of 3D7 VAR2CSA consisting of 442 peptides of 31mer each with an overlap of six residues. The peptides were bound to the chip using a cysteine at position 15. From the DBL1-3D7 immunizations, the original inhibitory serum sample was analysed on the chip. In addition sera from two rats from the subsequent immunizations, which did not induce inhibitory antibodies (Figure 3A), were analysed. In all the sera, there was a high reactivity against eight to 17 peptides of the total 63 peptides covering the DBL1 domain and three major B-cell epitopes were detected. Two of these epitopes reacted with IgG from all three sera, of which one was located between subdomain S1 and S2 (marked with a blue horizontal bar in figure 3A) and the other one at the beginning of subdomain S2 (orange in figure 3A), whereas the third epitope was predominantly recognized by the inhibitory serum and located in subdomain S3 (red horizontal bar, figure 3A). For DBL3-HB3 T1 the IgG reactivity from two rat sera were analysed, which inhibited parasite binding, and one rat serum that did not inhibit binding (Figure 3B). Two regions at the beginning of the S2 and S3 subdomain, respectively, were targeted by IgG from all three sera. However, only one peptide within each region was recognized by the non-inhibitory serum. The inhibitory DBL4-FCR3 antibodies published recently [21] were also mapped for epitopes using the same peptide array. It was difficult to identify a DBL4-FCR3 serum sample which did not induce inhibitory antibodies, therefore, a non-inhibitory DBL4-3D7 serum sample was chosen for comparison in the pepscan analysis (Figure 3C). Similar to the antibody reactivity induced against DBL1 and DBL3, a B-cell epitope was identified in the N-terminal part of the domain spanning the S1 and S2 subdomain border (orange horizontal bar in figure 3C) as well as a B-cell epitope in S3 (blue horizontal bar in figure 3C). All identified B-cell epitope regions were mapped on a model of the corresponding domain (Figure 3A-C).

## Discussion

Identification of the *var2csa *gene is one of the major recent advances in the search for a vaccine against PAM. The low level of polymorphism in VAR2CSA, relative to other PfEMP1 molecules [25], and the rapid acquisition of VAR2CSA-specific IgG as a function of parity after natural infections [13], support the potential of developing parts of this antigen into a vaccine. PAM pathogenesis is partly due to host immune responses in the placenta [26], and it may not be optimal to induce IgG that are not adhesion-inhibitory, since this may enhance unwanted inflammatory immune responses in the placental tissue. IgG that hinder parasite binding would reduce the harmful inflammatory response in the placenta and depending on the IgG isotype, these antibodies could also be efficiently opsonising. The strategy for vaccine development is therefore to induce anti-adhesive antibodies [27]. It has previously been shown that the DBL4 domain from FCR3 elicits a strong inhibitory IgG response, which was highly reproducible in subsequent immunizations [21]. This has raised the question whether DBL4 is the only part of VAR2CSA involved in CSA adhesion, and if other domains are indeed involved in the adhesion process, and accordingly it would be of interest to define these to make a multi-domain vaccine. Interestingly, in this study it is shown that two domains different from DBL4 can also elicit inhibitory antibodies. This indicates that the CSA binding region of VAR2CSA is comprised of epitopes from multiple domains, which is in line with published data showing that single recombinant domains from VAR2CSA do not bind specifically to CSA [15,17]. DBL4 is the most conserved VAR2CSA domain; however this domain does contain some variable regions, primarily located in the flexible loops [28]. At present, it is not known whether the anti-DBL4 IgG can inhibit parasite adhesion of all VAR2CSA expressing isolates. Therefore, a VAR2CSA vaccine may be comprised of DBL4 from different *var2csa *genes or FCR3 DBL4 and another DBL domain. From a vaccine development point of view, it is thus promising that it is possible to induce highly inhibitory antibodies with other domains than DBL4-FCR3. It is surprising though that a strong inhibitory antibody response could not be induced with the DBL4 domains from 3D7 and the two variants of HB3. In addition, the induced inhibitory response towards DBL3-HB3 T1 and DBL1-3D7 appeared to be difficult to reproduce in subsequent immunizations.

Based on the above findings, it is suggested that antibodies can block a single unique CSA binding-site present in the quaternary structure of VAR2CSA, or block the assembly of such quaternary structure to interfere with binding, and that these antibodies can be induced by a number of different VAR2CSA domains. This hypothesis is in line with previously published data suggesting that VAR2CSA is presented as a globular protein on the surface of IE [23]. However, it seems difficult to focus the immune response in the animals towards the correct epitopes, which is substantiated by the fact that the inhibitory response is either lost during the immunization schedule, or not induced at all despite the presence of high reactivity in ELISA.

The binding inhibitory activity was not induced in all immunizations; despite the presence of a specific and high titered immune response. This could be due to an erratic animal response to the immunogen caused by an unstable tertiary structure of the antigens used for immunizations. Another explanation could be differences in the fine specificity of the sera affecting the functionality of the induced IgG response, due to selection for reactivity against loop regions with high sequence variation, which this study would be sensitive to, since heterologous responses are measured. To study the fine specificity of the different sera, the IgG reactivity against VAR2CSA peptides was measured. In general, it was noted that the measured antibody reactivity against the different domains located to the same regions, and that a large central region of the domain did not contain any continuous epitopes. Interestingly, the large epitope in DBL3 located in a flexible loop is identical to the loop region shown to become structured upon CSA interaction [29]. DBL domains are compact and highly structured proteins and it is likely that many antibodies will be targeting discontinuous epitopes and thus would not be detected in the peptide array. However, it is clear from the data that different immunizations with the same antigen do not give the same fine specificity each time, which likely reflects the variation in the inhibitory activity of the induced antibodies.

## Conclusions

VAR2CSA consists of six DBL domains. The data presented here show that it is possible to induce parasite adhesion-blocking antibodies when immunizing animals with the domains DBL1 and DBL3, which indicates that the CSA binding site in VAR2CSA is comprised of regions from different domains. However, a robust and reproducible response with these proteins was not inducible. Epitope mapping shows differences in the fine specificity of the inhibitory and non-inhibitory sera. Accordingly if the *Baculovirus *produced DBL1-3D7 and DBL3-HB3 T1 antigens are to be used in a vaccine, further research is needed to define and focus the immune response towards the relevant epitopes.

## Abbreviations

CSA: chondroitin sulfate A; CSPG: chondroitin sulphate proeoglycan; DBL: Duffy-binding-like; EBA: Erythrocyte Binding Antigen; IE: infected erythrocytes; ELISA: Enzyme-Linked Immunosorbent Assay; IBA: Inhibition binding assay; IFA: Immunofluorescence assay; IgG: immunoglobulin gamma; PAM: pregnancy-associated malaria; PfEMP1: *Plasmodium falciparum *Erythrocyte Membrane Protein 1.

## Competing interests

The authors declare that they have no competing interests.

## Authors' contributions

AS and MD produced the proteins and MR and SBD immunized animals. MAN and VP performed the parasite experiments. MR, MAN, TM, MD, TGT and AS were responsible for the study design and the interpretation of the data. All authors contributed to writing of the manuscript and approved the final version.
